# Propensity Score Methods for Confounding Control in Observational Studies of Therapeutics for COVID-19 Infection

**DOI:** 10.1093/cid/ciae516

**Published:** 2024-10-16

**Authors:** Kathleen E Hurwitz, Nuvan Rathnayaka, Kayla Hendrickson, M Alan Brookhart

**Affiliations:** Epidemiology, Target RWE, Durham, NC, USA; Epidemiology, Target RWE, Durham, NC, USA; Epidemiology, Target RWE, Durham, NC, USA; Department of Population Health Sciences, Duke University, Durham, NC, USA

**Keywords:** observational studies, propensity score matching, COVID-19, inverse probability of treatment weighting, confounding

## Abstract

The authors provide a brief overview of different propensity score methods that can be used in observational research studies that lack randomization. Under specific assumptions, these methods result in unbiased estimates of causal effects, but the different ways propensity scores are used may require different assumptions and result in estimated treatment effects that can have meaningfully different interpretations. The authors review these issues and consider their implications for studies of therapeutics for coronavirus disease 2019.

Observational studies of the safety and effectiveness of therapeutic interventions are playing an increasingly important role in clinical medicine, drug regulation, and policy making [1]. Unlike randomized controlled trials (RCTs), which are often conducted in highly controlled environments with strict inclusion criteria, observational studies reflect the effects of treatments as they are used in routine clinical practice in large, heterogeneous populations of patients. The large size of these studies and their inclusivity allow for the study of rare safety outcomes and the estimation of treatment effects across many clinically relevant subgroups of patients that may be underrepresented in clinical trials.

The need for observational studies was particularly urgent during the global coronavirus disease 2019 (COVID-19) pandemic. Under the dual pressures of rising case numbers and spreading misinformation, many RCTs were conducted on potential treatments for outpatient and hospitalized COVID-19 patients [2]. Treatment guidelines were developed and updated at an unprecedented pace, and clinicians struggled to make evidence-based decisions within a disordered and evolving landscape of data. Vulnerable populations, such as organ transplant recipients, cancer patients, and pregnant women, were excluded from the initial wave of research, leaving clinicians treating those populations with little evidence-based guidance [3–5].

Many sources of bias that have historically plagued observational studies are well understood and can be avoided with principled study design [6, 7] that can be thought of as an attempt to emulate a randomized trial [8]. However, in an observational study, randomization is absent and patient risk factors are often imbalanced across treatment groups. Thus, any observed association between treatment and outcomes may be a result of differences in prognosis between the groups, rather than any causal effect of the treatment [9]. The problem of confounding must be addressed with statistical modeling, often using propensity scores. Propensity score methods make specific assumptions and can result in treatment effects that generalize to different patient populations. Proper assessment and interpretation of studies using propensity scores require an understanding of these issues. In this commentary, we review several propensity score–based methods, describe the assumptions they make, and describe the target populations of the estimators. We consider these issues in the specific setting of studies of treatments for COVID-19.

##  

### What Is a Propensity Score?

The propensity score is the conditional probability of being treated given a set of baseline covariates that are sufficient to control confounding. In practice, the propensity score is estimated using a statistical model of treatment assignment given a set of baseline covariates that are believed to confound the treatment–outcome relationship of interest. Causal inference with propensity score methods relies on several assumptions, including (1) *conditional exchangeability*, which requires that among people with the same confounders, treatment choice is unrelated to the outcome; (2) *no interference*, which requires that one person's treatment will not affect another person's outcome [10]; and (3) *correct model specification*, which states that the statistical model for the propensity score correctly represents the relations among the covariates. All approaches that aim to estimate causal effects also assume that the intervention being studied is well defined and can be identified in the data [11, 12].

### When Is the Assumption of Conditional Exchangeability or No Unmeasured Confounding Reasonable?

Confounding in observational studies can arise from numerous sources. In studies of intended effects (vs safety), confounding by indication or disease severity is often the primary concern [13]. This type of confounding results from good medical practice—physicians prescribing more intensive treatments to patients perceived to be at higher risk. Confounding can also arise from differences in frailty, access to care, cognitive status, or the channeling of patients away from medication that may pose specific risks to them [9].

Controlling confounding requires subject matter knowledge, and we cannot know from the data alone whether confounding is fully controlled [14, 15]. Therefore, it is crucial to understand how medications are used and any other external factors that may influence their use. This requires analysts to work closely with clinical and policy experts to elicit this information and identify a minimal set of adjustment variables that must be included in a propensity score model [16]. In some cases, proxies for unmeasured variables may be used in place of the variables themselves [17, 18].

Often this subject matter knowledge is depicted using directed acyclic causal graphs (DAGs) [16, 19]. In these graphs, the arrows (or edges) depict assumed causal relations. In Figure 1, we depict an example DAG, for a study of remdesivir and inpatient mortality among patients hospitalized with COVID-19. This DAG depicts secular changes in both the use of remdesivir and dexamethasone, as well as changes in the prevailing variant. The prevailing variant is assumed to affect disease severity, and supplemental oxygen requirement is depicted as a proxy for disease severity. Under this DAG, it would be sufficient to include both supplemental oxygen requirement and calendar time in a propensity score model to obtain an unbiased estimate of the effect of remdesivir on in-hospital mortality.

**Figure 1.**
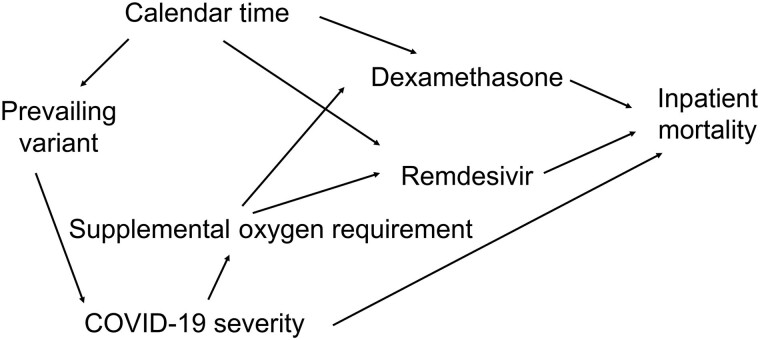
Directed acyclic graph for a hypothetical study of remdesivir treatment and inpatient mortality among patients hospitalized for coronavirus disease 2019 (COVID-19).

Although the absence of confounding is untestable and empirically unverifiable, it is critical to describe the sociodemographic and clinical characteristics of each treatment group at baseline. This can help the researcher understand the nature and extent of the confounding that may exist due to measured variables. If the groups are very different, it may suggest that unmeasured factors are likely out of balance as well. Any subsequent comparative analyses would be at great risk for bias. We can also examine covariate balance after propensity score adjustment to see how well we have controlled confounding. The most common approach for evaluating balance between treatment groups is the standardized mean difference (SMD) [20]. The SMD is a measure of difference between 2 means that is not dependent on sample size (unlike *P* values). If, after adjustment, the absolute SMDs are >0.1, the propensity score model may have to be modified to achieve better balance. However, even if the propensity score model achieves balance on measured covariates, there is no guarantee that unmeasured covariates will also be balanced.

Confounding can also be minimized with careful study design that ensures the treatments being compared are used for the same indications or restricting the study eligibility to the patient population to those who would be using the medications for the same indications [21]. For example, in the setting of COVID-19, it would be difficult to compare remdesivir to nirmatrelvir-ritonavir given their substantially different indications. According to guidelines issued by the National Institutes of Health and World Health Organization and the medications' labels, nirmatrelvir-ritonavir is intended to prevent hospitalization in high-risk patients with mild COVID-19 in an outpatient setting. In contrast, remdesivir is intended to prevent mortality in hospitalized patients who require some level of oxygen support. Therefore, comparative analyses of remdesivir and nirmatrelvir-ritonavir would be expected to to be subject to substantial confounding by indication. This should be evident in the “Table 1 of a research manuscript or output.” data through higher prevalence of markers of disease severity in the remdesivir group. The confounding by indication could be addressed by restricting the analysis to those patients who might be candidates for either treatment, if such a subpopulation existed.

**Table 1.** Summary of the Assumptions and Interpretations of Effect Estimates Produced by Different Propensity Score–Based Estimators

Estimator	Interpretation of Effect Estimates	Additional Assumptions	Example
Inverse probability of treatment weighting	Average effect of treatment in the population (ATE). Same treatment effect that would be estimated in a randomized trial with the same I/E criteria as the observational study.	Requires a positivity assumption that asserts that all patients have some possibility of being treated with any of treatments under study.	The effect of treatment with remdesivir vs dexamethasone alone in all patients hospitalized during the study period meeting I/E criteria.
Standardized mortality ratio weighting	Average effect of treatment in the treated (ATT). This estimator attempts to determine how well treatment worked among the patients who were treated. This weighting scheme is often used to reweight external control arm data to patients in a single-arm trial.	Requires a reduced positivity assumption that asserts that all patients who are treated have some possibility of having been untreated. Requires that all treated patients have comparable untreated patients.	The effect of treatment with remdesivir vs dexamethasone alone in the patients treated with remdesivir during the study period. The estimand will depend on who was treated with remdesivir and when it was used. For example, if the use and effectiveness of remdesivir differs by variant era, the ATT could be meaningfully different from the ATE.
Overlap weighting	Average effect of treatment in the “overlap population,” which is often described as the population where the most clinical equipoise may exist. This population cannot be described with I/E criteria but may be characterized in a Table 1 of a research manuscript or output.	This estimator does not target an average treatment effect in an *a priori* defined population, so therefore does not require positivity assumption.	The effect of treatment with remdesivir vs dexamethasone alone in the patients where there appears to be the most equipoise between these 2 treatment options. The population should be described in a Table 1 of a research manuscript or output.
Propensity score matching	Average effect of treatment in the population represented by the matched sample. In situations where all treated patients can be matched to untreated patients, this estimator will target the ATT. The population to which this estimator generalizes cannot be described with I/E criteria but may be characterized in a Table 1 of a research manuscript or output.	This estimator does not explicitly target an average treatment effect in an *a priori* defined population, so does not require a positivity assumption. If the reduced positivity assumptions holds, the estimator would target the ATT.	If all remdesivir patients can be matched to dexamethasone patients, this estimator would result in an estimate of the ATT and would have the same interpretation as the SMRW estimator . If not all remdesivir patients can be matched, the estimator would be more difficult to interpret. The population should be described in a Table 1 of a research manuscript or output.

Abbreviations: ATE, average effect of treatment in the population; ATT, average effect of treatment in the treated; I/E, inclusion/exclusion; SMRW, standard morbidity ratio weighted.

One common approach to investigate whether residual confounding may be present is through an analysis of negative control outcomes (NCOs) [22–24]. An NCO is a variable thought to share the same confounding structure as the outcome of interest but is not causally related to treatment. For example, in studies of influenza vaccines among the elderly and patients on hemodialysis, mortality before the start of the influenza season has been used as an NCO to detect bias [25, 26]. In both examples, the influenza vaccine was found to be strongly protective against mortality before influenza was present in the community, suggesting strong residual confounding. In addition to an NCO analysis, various types of sensitivity analysis can be performed to assess the extent to which uncontrolled confounding may explain away any observed effect of treatment [27, 28].

Last, although confounding is usually the primary concern in most observational studies, often researchers need to address problems with missing data, informative censoring, and measurement error. These issues can be minimized by ensuring data are fit-for-purpose [29], with remaining problems handled through a combination of carefully considered assumptions and statistical modeling [30–32].

### How Are Propensity Scores Used, and How Do They Alter the Interpretation of Results?

Propensity scores can be used in a variety of ways to estimate causal effects. In this article we focus on the use of propensity score estimators based on matching and weighting. For more extensive reviews of propensity score and related methods, we refer the reader to one of the many review papers at varying levels of technicality [33–36].

Inverse probability of treatment weighted (IPTW) estimation uses the propensity score (PS) to estimate the average effect of treatment in the population represented by the sample. This is the same parameter that would be estimated if one were to do a randomized trial. In an IPTW analysis, everyone is weighted by the inverse of the probability of receiving the treatment they received. In a study where some individuals are treated and others are untreated, treated individuals would receive a weight equal to 1 / PS, and patients who are untreated would receive a weight of 1 / (1 – PS). This approach upweights individuals who receive an unlikely treatment, since they are effectively underrepresented in their treatment group. Estimation of an average treatment effect also requires a “positivity” assumption, which requires all individuals to have a nonzero or positive probability of being treated with any treatment under study. Near violations of positivity can result in very large weights and imprecise parameter estimates. This partly reflects the fact that the data are not informative about the effects of treatment in patients who are rarely treated or who are almost always treated. More precise estimates may be obtained using augmented IPTW estimators. Alternatively, when weights are highly variable, the population may be trimmed by dropping those observations with very large or small propensity scores [37, 38]. While this may improve the variance of the estimated effect, it results in an obscured target population [38, 39]. In some situations, it may be possible to alleviate such positivity problems by simply making changes to the target population; for example, by excluding some subgroups that are almost always or never treated [40]. This changes the target population, but unlike trimming, the population can be clearly described and thus the findings of the study are more readily interpreted.

Standard morbidity ratio weighted (SMRW) estimation uses the propensity score to estimate the effect of treatment among the group of patients who received treatment [41]. This is often called the average effect of treatment in the treated (ATT) and is the parameter that would be estimated if one were to do an RCT in the population represented by the patients who were treated. SMRW estimation gives all treated individuals a weight of 1 and all untreated individuals a weight of PS / (1 – PS). This approach upweights the patients in the untreated group who most resemble treated patients and downweights those who are dissimilar. Similar to IPTW, SMRW estimation requires all treated patients to have a nonzero probability of being untreated. Intuitively, if there are types of patients in the treated arm who do not exist in the untreated group, the data are not informative about the effects of treatment in those patients. In the reweighted sample, the covariate distribution in the untreated sample resembles the distribution among treated patients. SMRW estimation can be generalized to more than 2 groups by reweighting all groups to resemble 1 selected group.

SMRW estimation is often used to analyze or contextualize data from single-arm trials with an external control arm [42, 43]. Since the external control arm may be drawn from a different population, SMRW approaches can be used to make the external control arm patients more comparable to those in the trial.

Overlap weighted (OW) estimation reweights the population so that patients in the region of the propensity score distribution with the most overlap between treatment groups are upweighted [44]. The overlap weight is the probability that an individual is assigned to the opposite group. Thus, treated patients are weighted by the probability of not receiving treatment (1 – PS), and untreated patients are weighted by the probability of receiving the treatment (PS). The weights are then normalized to sum to 1 within each treatment group. OW estimation was proposed as an approach to address settings in which individuals are rarely treated or untreated resulting in near violations of the positivity assumption made by IPTW estimation. With OW, there is no requirement that everyone in a specified population has some possibility of treatment because the estimator does not target a treatment effect in an *a priori* defined population. Therefore, this approach would effectively exclude patients who are rarely treated or untreated (with propensity score of 0 or 1). The population targeted by OW estimation depends on the distribution of the propensity scores [45]. This population cannot be precisely described (eg, with a set of inclusion and exclusion criteria) but is often described as the population where clinical equipoise happened to exist. If one conducted the same study in an identical population, but where the use of the treatment was different (eg, in a different era), it would be possible to obtain a meaningfully different treatment effect estimate.

Propensity score matching is an intuitive and commonly used approach that involves matching treated patients to 1 or more untreated patients based on the propensity score. Unmatched treated and untreated patients are discarded and not further analyzed. The matched sample is then analyzed as if the data were obtained from an RCT. Matching is effective at balancing covariates between the treatment groups. However, like OW estimation, the resulting parameter estimate does not generalize to an *a priori* defined population. Instead, the target population to which the estimate generalizes emerges from the analysis. In some, but not all cases, all treated patients can be matched to untreated patients, thus yielding an estimator of the ATT. If positivity holds, all treated patients should be matchable, at least in large samples, and the propensity score–matched estimator should target the ATT.

In the setting of treatments for COVID-19, the various propensity score–based methods discussed here may yield meaningfully different estimates of treatment effects, even if measured confounding is fully controlled. During the pandemic, there were significant changes over time in clinical practice, the prevailing variant, and the types of patients being hospitalized. In Figure 2, we depict the changes in the use of remdesivir and dexamethasone during the COVID-19 pandemic in a large cohort of patients hospitalized with COVID-19. If one were to conduct a study comparing the early use of remdesivir during hospitalization for COVID-19 with or without dexamethasone vs dexamethasone alone, it is reasonable to expect that the relative effectiveness of these therapies could differ over time. This could be due to the evolution of the virus that may alter its virulence or susceptibility to treatment, increasing prevalence of vaccination and natural immunity in the population, changing aspects of the patient case-mix, or other changes to the standard of care for patients hospitalized for COVID-19. In Figure 2, we see that remdesivir was used more frequently during the Delta variant period than either before or after; therefore, an IPTW estimator would upweight patients in the pre-Delta and Omicron periods and downweight those patients treated with remdesivir during the Delta period. An IPTW estimator in this setting would try to answer the question, what would we have seen had we conducted an RCT in this population across all the variant eras? It would provide an estimate of what would have happened if everyone hospitalized during the pandemic had been treated with remdesivir compared to what would have happened if everyone had been treated with dexamethasone alone. In contrast, the SMRW estimator would attempt to determine how well treatment worked in the group of patients who were treated. Hypothetically, if remdesivir (compared to dexamethasone alone) was more effective in the pre-Delta era, the SMRW estimator would result in a smaller estimate of benefit than the IPTW estimator given the decreased effectiveness of remdesivir among patients treated later in the pandemic. If all remdesivir patients could be matched to dexamethasone patients, propensity score matching would yield an estimator of the ATT, like the SMRW estimator. Otherwise, the target population could only be characterized by the distribution of the patient characteristics. Likely, OW would downweight patients early in the pandemic, when fewer eligible patients were treated with remdesivir. The relative weighting of patients later in the pandemic would depend on the distribution of the propensity score in both treatment groups. Patients with a propensity score closest to 0.5 would receive the largest weights. The assumptions and interpretations of different propensity score estimation approaches are summarized in Table 1.

**Figure 2.**
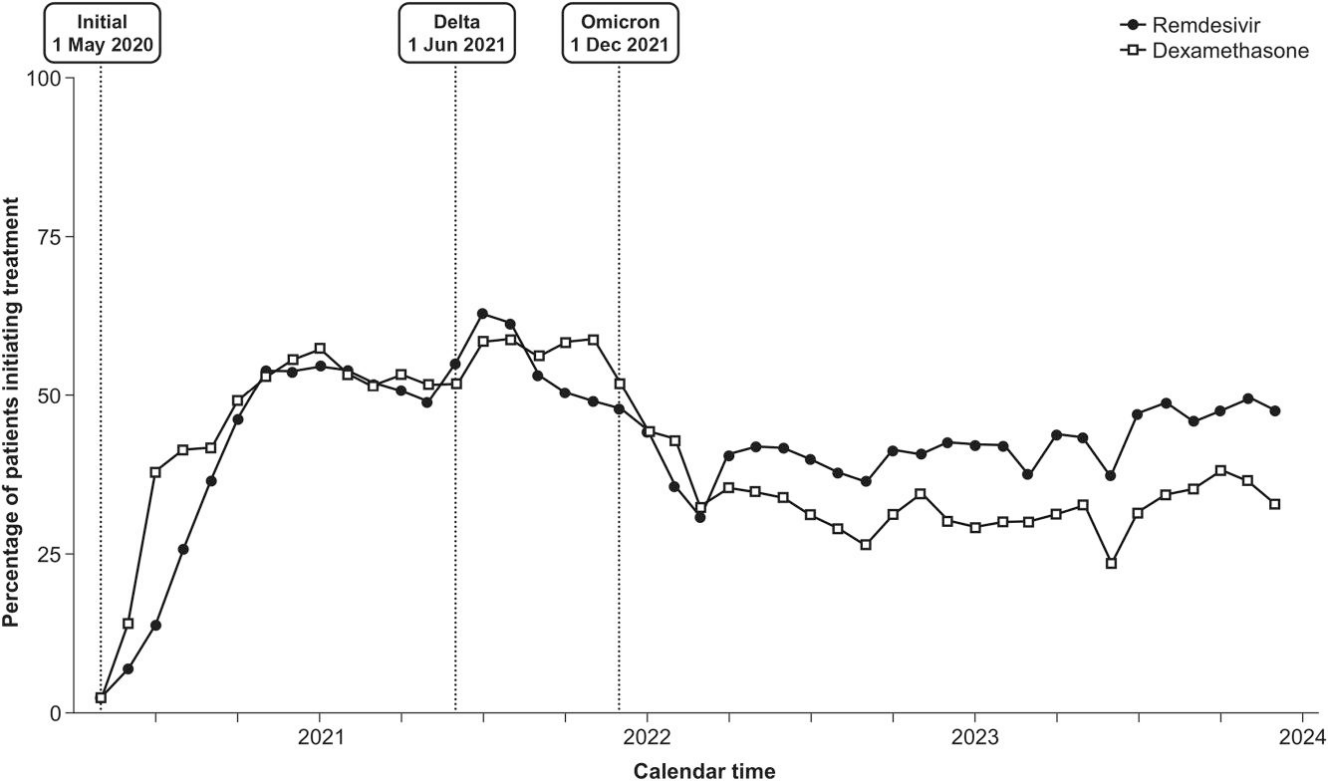
Change in the use of remdesivir and dexamethasone during the coronavirus disease 2019 pandemic.

## CONCLUSIONS

Observational research is likely to play an increasingly important role in developing treatment guidelines for understudied populations, complex patients, and rare diseases. The COVID-19 pandemic highlighted the need for timely and high-quality real-world evidence. As COVID-19 has progressed to endemicity, it is unlikely that additional RCTs will be conducted that will help determine optimal treatment regimens, leaving the clinical community increasingly reliant on observational research with real-world data. For clinicians and policy makers to identify best practices from observational studies, it is crucial to understand how to properly evaluate and interpret the results of these studies. There are many approaches to and aspects of observational research that affect the validity and interpretability of results. In this commentary, we have discussed key assumptions behind different propensity score–based approaches and how the different approaches can result in estimated treatment effects with meaningfully different interpretations.

## Notes

***Author contributions.*** All authors contributed substantially to the manuscript and agreed to the final submitted version.

***Financial support***. This work was supported by Gilead Sciences, Inc.

***Supplement sponsorship.*** This article appears as part of the supplement “Harnessing Real-world Data to Inform Clinical Practice: Lessons From the Evolving COVID-19 Era,” sponsored by Gilead Sciences, Inc.

***Potential conflicts of interest***. K. E. H., N. R., and K. H. are employees of and own equity in Target RWE. M. A. B. serves on scientific advisory committees for the American Association for Allergy Asthma and Immunology, Amgen, Brigham and Women's Hospital, Gilead Sciences, Intercept, National Institute of Diabetes and Digestive and Kidney Diseases, Target RWE, and Regeneron; he also has equity with AccompanyHealth, Target RWE, and VitriVax.
